# Candida thrombophlebitis in children: a systematic review of the literature

**DOI:** 10.1186/s13052-020-00913-5

**Published:** 2020-10-06

**Authors:** Claudia Colomba, Luigi Campa, Lucia Siracusa, Salvatore Giordano, Maria C. Vella, Giovanni Corsello, Mario Giuffrè, Antonio Cascio

**Affiliations:** 1 Department of Health Promotion, Maternal and Infant Care, Internal Medicine and Medical Specialties, University of Palermo, Palermo, Italy; 2 Pediatric Infectious Diseases Unit, “G. Di Cristina” Hospital, ARNAS Civico, Palermo, Italy

**Keywords:** Thrombosis, Phlebitis, Children, Newborn, Candida, Sepsis, Hospital-acquired infections, Persistent fever, Enoxaparin, Antifungal therapy

## Abstract

**Objective:**

To describe a case of thrombophlebitis associated with Candida infection and to analyze other published reports to define clinical characteristics, prognostic data, diagnostic and therapeutic strategies.

**Study design:**

A computerized search was performed without language restriction using PubMed and Scopus databases. An article was considered eligible for inclusion if it reported cases with Candida thrombophlebitis. Our case was also included in the analysis.

**Results:**

A total of 16 articles reporting 27 cases of Candida thrombophlebitis were included in our review. The median age of patients was 4 years. In 10 cases there was a thrombophlebitis of peripheral veins; in the remaining cases the deep venous circle was interested. *Candida albicans* was the most frequently involved fungal species. The most recurrent risk factors were central venous catheter (19/28), broad spectrum antibiotics (17/28), intensive care unit (8/28), surgery (3/28), mechanical assisted ventilation (5/28), total parenteral nutrition (8/28), cancer (2/28), premature birth (6/28), cystic fibrosis (2/28). Fever was the most frequent clinical feature. All children with peripheral and deep thrombophlebitis were given antifungal therapy: amphotericin B was the most used, alone or in combination with other antifungal drugs. Heparin was most frequently used as anticoagulant therapy. Illness was fatal in two cases.

**Conclusion:**

Candida thrombophlebitis is a rare but likely underdiagnosed infectious complication in pediatric critically ill patients. It is closely connected to risk factors such as central venous catheter, hospitalization in intensive care unit, prematurity, assisted ventilation, chronic inflammatory diseases. Antifungal therapy and anticoagulant drugs should be optimized for each patient and surgical resection is considered in the persistence of illness.

## Introduction

Suppurative thrombophlebitis complicating intravenous therapy is a rare but important health-care associated infection. It is well described in adult patients but unfrequently reported in children [1]. It has most often been associated with *Staphylococcus aureus* or coagulase-negative Staphylococci, even if the incidence of Candida infections is increasing [2]. Candida thrombophlebitis can affect the superficial or deep venous circulation. The latter is an even rarer event, reported more often in adult people. Central venous catheters (CVCs) represent an important predisposing factor, especially in combination with total parenteral nutrition (TPN) and multiple antibiotic therapy [3]. Clinical signs suggestive of suppurative peripheral thrombophlebitis are persisting inflammatory signs at a previous site of an intravenous catheter insertion, complicated by systemic symptoms including high fever and malaise. Instead, diagnosis of deep Candida thrombosis may be difficult and there must be a very high level of awareness whenever a patient has the risk factors for Candida septic thrombophlebitis [1, 3].

Peripheral thrombophlebitis often responds well to catheter removal, local drainage, short course of antifungal therapy and surgical excision of the thrombosed part of the vein is recommended [4].

Central venous infection poses a greater problem, however, due to the formidable undertaking in surgical remove of the clot from the central veins and thrombophlebitis can be successfully managed by removal of the catheter, antifungal therapy, and systemic anticoagulation therapy [3, 4].

### We describe here the case of a child who developed thrombophlebitis of the iliac-femoral axis by *Candida albicans*

The aim of our study is to focus attention on Candida thrombophlebitis in children and to analyze the epidemiologic and clinical characteristics of Candida thrombophlebitis in pediatric patients through a systematic review of the literature.

## Case report

A 2-year-old girl with a recent history of recurrent respiratory infections was admitted to the “G. Di Cristina” Children’s Hospital in Palermo, Sicily, Italy, for fever and cough. Upon admission, she had mild dyspnea with polypnea (respiratory rate 40/min), with bilateral basal crackles in both lungs. Blood tests revealed white blood cell 9.810/mm^3^ (neutrophils 40%, leukocytes 55%) and C-reactive protein 2,66 mg/dl. A chest X-ray revealed pneumonia. Treatment with ceftazidime (100 mg/kg/die) was started with resolution of fever after 3 days. Ten days later, she presented fever and clinical worsening, respiratory distress, onset of pneumothorax and subcutaneous emphysema involving the upper limb and neck, the patient was transferred to the ICU and a central venous catheter was placed in the left femoral vein. Seven days after, the patient developed continuous fever (higher temperature 40 °C) and CVC was removed. Blood and CVC cultures were positive for azole-resistant *Candida albicans*. The patient was transferred to the Infectious Disease Unit where a treatment with amphotericin B (3 mg/kg/die) and caspofungin (70 mg/m^2^/die in first day, then 50 mg/m^2^/die) was started. For the persistence of fever and onset of edema of the lower limb, a chest and abdominal CT scan was performed with evidence of thrombophlebitis of left iliac-femoral axis and associated fistula of paravertebral and gluteal muscles, confirmed by a subsequent thorax and abdomen MRI scan. Therapy with sub cutaneous enoxaparin was started (100 UI/kg). A sweat test was carried out, with positive result, but subsequent genetic study excluded mutations associated to cystic fibrosis.

After a week, fever persisted and a CT scan showed no improvement of the deep vein thrombosis extension. The child was transferred to another hospital unit, specialized in thrombosis in pediatric age, where treatment was implemented with prednisone administration. A gradual improvement of clinical and instrumental picture was achieved without surgical resection.

## Literature search

A computerized search was performed without language restriction using PubMed and Scopus, combining the terms (phlebitis OR venous thrombosis OR thrombosis) AND (candid* OR fungal*) AND (children OR child OR infant OR baby), with no filters. Furthermore, all references listed were hand-searched for other relevant articles. An article was considered eligible for inclusion if it reported cases with full clinical data consistent with Candida thrombophlebitis. The following epidemiologic and clinical variables were evaluated for each case: sex, age, risk factors for candidemia, Candida species, clinical features, therapy and outcome.

The selected articles were reviewed by two independent authors, and judged on their relevant contribution to the subject of the study. The Preferred Reporting Items for Systematic Review And Meta-Analysis (PRISMA) guidelines were followed.

## Results

After an extensive search in MEDLINE, 355 articles were found; only 13 of the above papers were selected for inclusion [5–17]. Three additional papers not present in Medline were added after a hand search of the bibliography of the above papers [18–20].

**Fig. 1:**
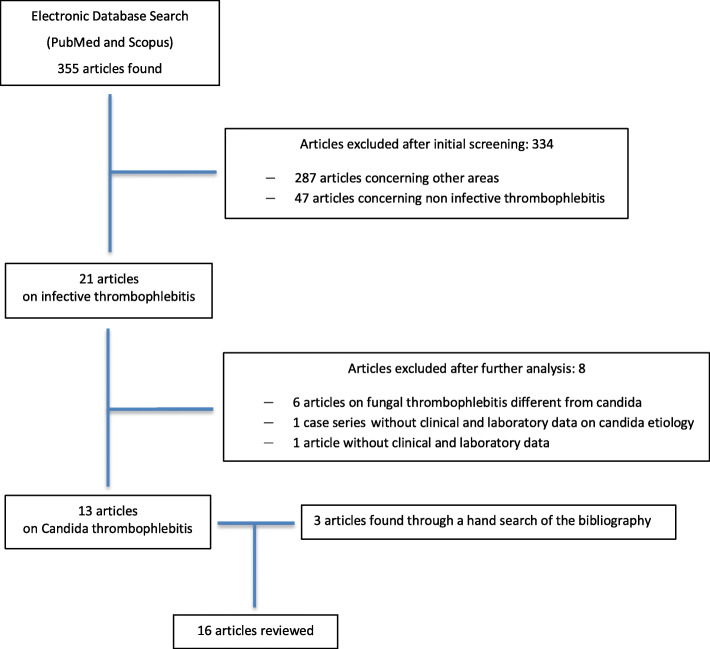
Flow chart summarizing the literature search approach

A total of 16 articles reporting 27 cases of Candida thrombophlebitis were included in our review. A flow chart summarizing the literature research approach is reported in Fig. 1. Most of the articles were single case reports.

We analyzed anamnestic and clinical data of 28 patients (including our new case). Risk factors, clinical features, diagnosis, therapy and mortality have been analytically reported in Table 1.

**Table 1:** Data from the reported cases of Candida thrombophlebitis in pediatric patients

Author/year/Country [Ref]	Age/Sex	Initial clinical conditions and/or related procedures	Risk factors	Candida species	Location	Presentation	Therapy	Outcome
Wiley/1977/USA [5]	PN / F	tracheoesophageal fistula, gastrostomy, cardiopulmunary arrests, aspiration pneumonia	CVC, TPN, ICU, surgery, AB	*C. albicans* *C. parapsilosis*	SVC, RV	edema of the face and neck	amfotericin B5, flucytosine	died
Miller/1981/USA [6]	18 mo / M	acute undifferentiated leukemia	CVC, cancer, CT, AB	C. albicans	axillary and subclavian vein	fever	antifungal therapy (n.s.)	survived
	10 y / F	acute myeloid leukemia	CVC, cancer	*C. tropicalis*	giugular vein	fever	antinfungal agents (n.s)	survived
Torres-Rojas/1982/USA [18]	11 y / F	acute rheumatic fever	AB, steroids	C. albicans.	n.s.	fever	amphotericin B, 5-fluorocytosine, excision of vein	survived
Malfroot/1986/Belgium [20]	17 y / M	CF, pneumonia	AB, CVC, advanced pulmonary disease	C. albicans	basilica vein	fever, phlebitis	amphotericin B, flucytosine, surgical excision	survived
Lacey/1988/USA [7]	13 mo/NR	broncopulmonary displasia, ECMO survivor	AB, TPN, CVC	C. albicans	IVC	sepsis, head and neck swelling	amphotericin B, 5 fluorocytosine, streptokinase	survived
	4 mo / NR	midgut volvulus	AB, TPN, CVC	C. tropicalis	SVC, IVC	sepsis, head and neck swelling	amphotericin B 5 fluorocytosine streptokinase	survived
	4 mo / NR	pentalogy of Cantrell	AB, TPN, CVC	C. albicans	SVC	sepsis, head and neck swelling	amphotericin-B, 5 fluorocytosine, streptokinase	survived
	11 mo / NR	Hirschsprung’s disease	AB, TPN, CVC	C. albicans	SVC, IVC	sepsis, head and neck swelling	amphotericin B, 5 fluorocytosine, streptokinase	survived
Cotton/1988/Rep. of South Africa [8]	PN / F	hyaline membrane disease	assisted ventilation, AB	C. parapsilosis	vein of the dorsum of the foot	phlebitis	amphotericin B, surgical excision	survived
Berg/1989/USA [9]	5 y / F	burns and surgical debridement, sepsis, pneumonia	wound infection, ICU, AB, CVC	C. albicans	iliac and femoral veins, IVC	sepsis, phlebitis	amphotericinB, flucytosine, heparin	survived
Ashkenazi/1990/USA [10]	4 mo / M	upper respiratory tract infection, respiratory distress (by RSV), double aortic arch	CVC, surgery, ICU, AB	C. tropicalis	IVC, renal vein	fever	amphotericin B, heparin	survived
Cherrick/1995/USA [11]	16 mo/M	neuroblastoma, sepsis	CVC, AB, TPN, CT	C. albicans	innominate vein	fever, respiratory distress	amphotericin B, flucytosine, streptokinase	survived
Friedland/1996/Rep. of South Africa [12]	6 mo / NR	diarrhea, sepsis	n.s.	C. parapsilosis	cubital vein	fever	fluconazole	survived
	3 mo / NR	diarrhea	malnutrition	C. parapsilosis	cubital vein	phlebitis	flucanazole, incision and drainage	survived
	7 mo / NR	pneumonia	n.s.	C. albicans	forearm vein	phlebitis	fluconazole	survived
	22 mo / NR	prematurity	prematurity	C. albicans	cubital	phlebitis	fluconazole, incision and drainage	survived
	4 mo / NR	diarrhea	n.s.	C. parapsilosis	hand vein	phlebitis	fluconazole, aspiration	survived
	8 mo / NR	diarrhea, HIV	HIV	C. parapsilosis	cubital vein	phlebitis	amphotericin B, aspiration	survived
	9 mo / NR	diarrhea	TPN	C. parapsilosis	cubital vein	phlebitis	amphotericin B, incision and drainage	survived
Garcia/1997/France [19]	14 y / F	ventricular tachycardia, cardiac arrest	ICU, CVC, MAV	C. albicans	left subclavian vein, right giugula, SVC, RA	fever	amphotericin B, flucytosine, heparin, caval thrombectomy	survived
Pacifico/2004/Italy [13]	PN / M	respiratory distress, prematurity	tri-twin pregnancy, prematurity, CVC, NICU, AB, MAV, maternal fever	C. albicans	right and main portal veins	abdominal distension	amphotericin B, fluconazole	survived
	PN / M	prematurity, maternal fever	prematurity, NICU, CVC, MAV, AB	C. albicans	main portal vein	fever, abdominal distension	amphotericin B	survived
Sharma/2009/USA [14]	PN / M	prematurity, patent ductus arteriosus, respiratory distress syndrome, intestinal perforation	prematurity, AB, surgery, CVC	C. albicans	RA, IVC	NR	liposomal amphotericin B, caspofungin, heparin	died
Giuffrè/2016/Italy [15]	PN / NR	prematurity	prematurity, TPN, UVC, intestinal occlusion, MTHFR gene mutation (C677T), homozygous PAI-1 gene polymorphism (4G/4G)	C. parapsilosis	umbilical vein and left branch of the hepatic portal vein	perihepatic abscess	drainage of the collection, removal of umbilical vessels, micafungin	survived
Manish/2017/India [16]	4 y / F	diabetic ketoacidosis	ICU, MAV, CVC	C. albicans C. tropical	right external iliac vein, common femoral, deep femoral vein	fever	fluconazole	survived
Schapkaitz/2019/Rep. of South Africa [17]	5 y/F	CF, respiratory infection, AB	CVC, AB	C. parapsilosis	endocarditis, SVC, RA	fever	amphotericin B, fluconazole	survived
present case/2020/Italy	2 y / F	respiratory infection, pneumothorax and subcutaneous emphysema	CVC, ICU, AB	C. albicans	left iliac-femoral axis	fever, edema of leg	liposomial amphotericin B, caspofungin, enoxaparin	survived

*CVC* Central venous catheter, *ICU* Intensive care unit; *NICU* Neonatal intensive care unit, *TPN* total parenteral nutrition, *AB* Antibiotic therapy, *SCV* Superior vena cava, *ICV* Inferior vena cava, *RV* Right ventricle

*RA* Right atrial, *CT* Chemotherapy, *CF* Cystic fibrosis, *RSV* Respiratory syncytial virus, *MAV* Mechanical assisted ventilation, *RV* Right ventricle, *UVC* Umbilical vein catheter, *n.s*. Not specified, *NR* Not reported, *PN* Preterm newborn

There were 7 boys and 9 girls, in the remaining cases the gender was not specified. The median age was 4 years. Six cases were reported in premature newborns. Fourteen children were under 24 months old. In 10 cases thrombophlebitis interested peripheral veins [8, 18, 20], in the remaining cases the deep venous circle.

*Candida albicans* was isolated in 18 cases*, Candida parapsilosis* in 9 and *Candida tropicalis* in 4*.* In two cases 2 different species were simultaneously present (in one case *C. albicans* and *C. parapsilosis*, in the other case *C. albicans* and *C. tropicalis*). Risk factors for candidemia and thrombosis included CVC (19/28, 67%), broad spectrum antibiotics (17/28, 60%), admission to the ICU (8/28, 28%), surgery (3/28, 10%), mechanical assisted ventilation (5/28, 17%), TPN (8/28, 28%), cancer (2/28, 7%), premature birth (6/28, 21%), cystic fibrosis (2/28, 7%). In all cases of peripheral thrombophlebitis, the thrombosed and infected veins appeared thickened with inflammation of the overlying soft tissue and fever was present in 3 cases [12, 18, 20]. Among patients with deep venous thrombophlebitis, 88% patients (16/18) were febrile at the time of diagnosis.

Caval thrombosis was suspected in most patients on the basis of head and neck swelling. In all cases diagnosis was highly suspected because of persistence of candidemia and a vascular catheter in place prior to the onset of candidemia. Diagnosis was confirmed with whole-body gallium 67 scintigraphy in 2 cases, with venogram in 3 cases, with echocardiogram in 4 cases, and with ultrasonography in 9 cases.

All children with peripheral and deep thrombophlebitis were given antifungal therapy: 19 patients were treated with amphotericin B, 11 of which with a combination of amphotericin B and 5-fluorocytosine, 2 with amphotericin B and echinocandin, one with amphotericin B and fluconazole and 5 with amphotericin B as monotherapy. Fluconazole has been used successfully on monotherapy in 6 cases. Liposomal amphotericin B was used only in another case apart from ours [14]. In 3/10 cases of the superficial thrombophlebitis, excision of the thrombosed part of the vein was necessary [8, 18, 20] and only in one case of deep thrombophlebitis the thrombus was surgically removed [19].

Data regarding anticoagulant therapy reported that heparin was used in 5 cases and thrombolytic therapy with continuous infusion of low-dose of streptokinase in others 5 cases of deep thrombophlebitis.

In two cases (both premature newborns) the outcome was fatal: one died because of a superior vena cava syndrome secondary to candida thrombophlebitis, the other one died because of renal failure secondary to inferior vena cava thrombosis with right atrial mycetoma. Both diagnoses were made at autopsy: in the first patient, the superior vena cava appeared obstructed throughout its length by an organizing thrombus and histology showed budding yeast and pseudohyphe [5]**,** in the second one the atrial thrombus revealed multiple hyphae mimicking a fungal ball [14].

## Discussion

Only 27 cases of Candida thrombophlebitis were retrieved after our accurate search. On the basis of the increasing incidence of nosocomial candidemia in children over the past decades and the high incidence of catheter-related thrombosis [21, 22], we would have expected to find many more cases. Therefore, we think Candida thrombophlebitis is likely an underdiagnosed or underreported disorder. Our search found that all but three cases were reported in three countries: USA, South Africa and Italy. Being Candida thrombophlebitis a health-care associated infection, we would expect it is more reported in countries with the highest incidence of hospital-acquired infections [23].

The pathogenesis of Candida thrombosis and related catheter-associated candidemia is not well understood. The source of candidemia has been a matter of debate, with some suggesting a skin origin and others a gut origin. Candidemia may arise from the gastrointestinal tract and portal venous circulation and enter the systemic circulation, leading to secondary infection of the vascular catheter [24, 25]. In this situation, the catheter is the target of fungemia rather than the source but it might continue to be the source of sustained candidemia as organisms proliferate on its surface. Vascular catheters, however, might serve as primary source of fungemia and infectious organisms may be introduced percutaneously along the catheter, or with contaminated intravenous solution [26, 27].

Certainly, the presence of a CVC is the most important risk factor for the development of deep venous thromboembolism in children. The reported incidence in pediatric patients ranges from 5 to 44% depending on age, the primary condition, and therapies received via the CVC, as well as the type and site of CVC placement [28]. Several possible mechanisms by which CVC causes thrombosis include damage to vessel wall, disrupted blood flow, infusion of substances that damage endothelial cells (in example in case of TPN), and thrombogenic catheter materials [29].

Antibiotic therapy, given in combination with TPN, is the most important risk factor for the development of candidemia, allowing fungal gut overgrowth with subsequent absorption through intact epithelium. The combination of the above risk factors, CVC, antibiotic therapy and TPN, exposes the patient to a very high risk of deep candida thrombophlebitis. All patients reported in this review had one of the described risk factors: more than 60% received antibiotic therapy for several days, 68% had prolonged venous catheterization and 28.6% received TPN.

Prematurity is an important risk factor for Candida thrombophlebitis. Low gestational age, low birth weight, immaturity of anatomical barriers, endotracheal intubation, mechanical ventilation, TPN, central venous catheterization, broad spectrum antibiotics treatment and surgery are considered the main risk factors for Candida spp. infection at birth [29]. All cases of neonatal thrombophlebitis in this review occurred in premature babies with more than 3 risk factors among those described above. The incidence of neonatal candidemia is reported as 2–6.8% among Very Low Birth Weight infants (less than 1500 g) and even higher in Extremely Low Birth Weight infants (less than 1000 g), ranging from 4 to 16%. The incidence increases with reduction of gestational age (inverse linear pattern) from around 3% at 28 weeks of gestation to 24% at 23 weeks of gestation [30, 31].

Transmission of Candida may be vertical (from the mother) or nosocomial, and umbilical vein catheter (UVC) is probably the most important risk factors. UVC positioning is a relatively easy procedure performed routinely in Neonatal Intensive Care Units. However, the possibility of an incorrect placement of the catheter may lead to a higher risk of complications, including thromboembolic complications and liver abscesses formation. The presence of a perihepatic abscess originated from a septic thrombus of the umbilical vein has been reported in literature and in our review [15, 31].

Our patient was investigated for cystic fibrosis, which is associated to a high frequency of acquired protein C and S deficiency as a result of vitamin K deficiency or associated liver involvement. Therefore, cystic fibrosis is a predisposing condition for venous thromboembolism, as occurred in 2 patients reported in this review [17, 20].

Deep candida thrombophlebitis is often occult and underdiagnosed; the diagnosis may be difficult, especially in the absence of metastatic phenomena [32] or localized edema and there must be a very high level of suspicion for the presence of risk factors. In our review, only patients with caval thrombosis showed head and neck swelling; in all other patients (61%) the occurrence or persistence of fever and a persistent candidemia after removal of a culture-positive CVC, despite effective antifungal treatment, suggested the presence of an intravascular focus of infection.

We suggest the opportunity of a routine check for deep thrombophlebitis on the basis of a poor catheter function in all children with chronic indwelling CVCs and septic signs. Eventually, echo-doppler and/or CT-scan of the central veins together with echocardiography should be considered for corroborating the definite diagnosis of deep thrombophlebitis [3].

Although radical excision of the affected veins in combination with antifungal therapy is the current recommendation in the treatment of peripheral Candida thrombophlebitis [4], in our review the excision of the thrombosed part of the vein was necessary only in 3 cases.

Few data are available concerning the best strategy for managing septic deep thrombophlebitis [1, 4]. In some cases, systemic anticoagulation or thrombolytic therapy has been used as adjunctive therapy, but there are insufficient data to recommend their use [4]. Removal of CVC, antifungal therapy and radiologic monitoring of the thrombus are recommended [4]. Unfortunately, the decision of removing a CVC in children is more difficult than in adults, due to the frequently limited pediatric intravascular access and to the risk of thrombus dislodgement and pulmonary embolization. Regarding antifungal therapy, lipid formulation of amphotericin B (L-AmB) (3–5 mg/kg daily), or fluconazole (6–12 mg/kg daily), or an echinocandin (caspofungin 70 mg/m^2^ as loading dose, then 50 mg/m^2^ daily, micafungin 2 mg/kg daily, or anidulafungin 1.5 mg/kg) for at least 2 weeks after candidemia (if present) has cleared is recommended for the treatment of Candida thrombophlebitis [4]. In our review amphotericin B deoxycholate (in combination with 5-fluorocytosine in more than half of the cases) was the most used drug. Fluconazole and caspofungin have also been successfully used in some cases. In particular conditions, such as in case of kidney failure, dosages of antifungal drugs should be modified according to the plasmatic levels of creatinine. Dosage interval of fluconazole may be extended up to 48–72 h also in neonatal age, depending on degree of prematurity. Among echinocandins, micafungin is registered for use in the first month of life (recommended dosage 7–10 mg/kg/die) and appears to be effective also against biofilm on intravascular catheters.

Sometimes medical therapy fails because the antifungal agents are unable to reach the infected site, as occurs with an abscess during an endovascular infection. Therefore, excision of the infected vein should be important but this approach is frequently not practical for most pediatric patients with deep Candida thrombophlebitis, due to the relative inaccessibility of the central veins and the high mortality and morbidity associated with surgical thrombectomy in these critically ill infants [3]. In our review a prolonged antifungal therapy and the removal of CVC, with or without anticoagulation, resulted in clinical cure in most cases.

In conclusion Candida thrombophlebitis is a rare but likely underdiagnosed infectious complication of the critically ill patients. Recurrent or persistent candidemia after removal of a contaminated CVC should raise the suspicion leading to key investigations for an early diagnosis. Prolonged fungicidal therapy with amphotericin B or echinocandins must be provided. Treatment should be optimized for each patient and surgical resection evaluated on the basis of perioperative risk.

## Data Availability

The datasets used and/or analyzed during the current study are available from the corresponding author on reasonable request.

## Abbreviations

CVC: Central venous catheter
ICU: Intensive care unit
TPN: Total parenteral nutrition
